# Comparison of nodal staging between CT, MRI, and [^18^F]-FDG PET/MRI in patients with newly diagnosed breast cancer

**DOI:** 10.1007/s00259-021-05502-0

**Published:** 2021-09-03

**Authors:** Janna Morawitz, Nils-Martin Bruckmann, Frederic Dietzel, Tim Ullrich, Ann-Kathrin Bittner, Oliver Hoffmann, Eugen Ruckhäberle, Svjetlana Mohrmann, Lena Häberle, Marc Ingenwerth, Daniel Benjamin Abrar, Lino Morris Sawicki, Katharina Breuckmann, Wolfgang Peter Fendler, Ken Herrmann, Christian Buchbender, Gerald Antoch, Lale Umutlu, Julian Kirchner

**Affiliations:** 1 Department of Diagnostic and Interventional Radiology, Medical Faculty, University Dusseldorf, Moorenstrasse 5, 40225 Dusseldorf, Germany; 2 Department of Gynecology and Obstetrics, University Hospital Essen, University of Duisburg-Essen, 45147 Essen, Germany; 3 Department of Gynecology, Medical Faculty, University Dusseldorf, 40225 Dusseldorf, Germany; 4 Institute of Pathology, Medical Faculty, Heinrich-Heine-University and University Hospital Duesseldorf, Duesseldorf, Germany; 5 Institute of Pathology, University Hospital Essen, West German Cancer Center, University Duisburg-Essen and the German Cancer Consortium (DKTK), Essen, Germany; 6 Department of Diagnostic and Interventional Radiology and Neuroradiology, University Hospital Essen, University of Duisburg-Essen, 45147 Essen, Germany; 7 Department of Nuclear Medicine, University of Duisburg-Essen and German Cancer Consortium (DKTK)-University Hospital Essen, Essen, Germany

**Keywords:** Nodal staging, Breast cancer, PET/MR

## Abstract

**Purpose:**

To compare CT, MRI, and [^18^F]-fluorodeoxyglucose positron emission tomography ([^18^F]-FDG PET/MRI) for nodal status, regarding quantity and location of metastatic locoregional lymph nodes in patients with newly diagnosed breast cancer.

**Materials and methods:**

One hundred eighty-two patients (mean age 52.7 ± 11.9 years) were included in this prospective double-center study. Patients underwent dedicated contrast-enhanced chest/abdomen/pelvis computed tomography (CT) and whole-body ([^18^F]-FDG PET/) magnet resonance imaging (MRI). Thoracal datasets were evaluated separately regarding quantity, lymph node station (axillary levels I–III, supraclavicular, internal mammary chain), and lesion character (benign vs. malign). Histopathology served as reference standard for patient-based analysis. Patient-based and lesion-based analyses were compared by a McNemar test. Sensitivity, specificity, positive and negative predictive values, and accuracy were assessed for all three imaging modalities.

**Results:**

On a patient-based analysis, PET/MRI correctly detected significantly more nodal positive patients than MRI (*p* < 0.0001) and CT (*p* < 0.0001). No statistically significant difference was seen between CT and MRI. PET/MRI detected 193 lesions in 75 patients (41.2%), while MRI detected 123 lesions in 56 patients (30.8%) and CT detected 104 lesions in 50 patients, respectively. Differences were statistically significant on a lesion-based analysis (PET/MRI vs. MRI, *p* < 0.0001; PET/MRI vs. CT, *p* < 0.0001; MRI vs. CT, *p* = 0.015). Subgroup analysis for different lymph node stations showed that PET/MRI detected significantly more lymph node metastases than MRI and CT in each location (axillary levels I–III, supraclavicular, mammary internal chain). MRI was superior to CT only in axillary level I (*p* = 0.0291).

**Conclusion:**

[^18^F]-FDG PET/MRI outperforms CT or MRI in detecting nodal involvement on a patient-based analysis and on a lesion-based analysis. Furthermore, PET/MRI was superior to CT or MRI in detecting lymph node metastases in all lymph node stations. Of all the tested imaging modalities, PET/MRI showed the highest sensitivity, whereas CT showed the lowest sensitivity, but was most specific.

## Introduction

With more than 2 million cases in 2020, breast cancer is the most commonly diagnosed cancer worldwide [1]. Despite tumor biology, nodal involvement is one of the most significant prognostic factors at initial diagnosis of breast cancer [2, 3]. For breast cancer, locoregional lymph nodes are defined as ipsilateral axillary, supraclavicular, and internal mammary lymph nodes [4].

The likelihood of lymphatic drainage from the breast to axillary, internal mammary, infraclavicular, and supraclavicular lymph nodes are reported to be 98.2%, 35.3%, 1.7%, and 3.1% [5], but the location of lymph node metastases significantly depends on primary tumor location [6]. All further affected lymph nodes are considered distant metastasis. Nowadays, staging for primary breast cancer patients consists of clinical examination, mammography, breast- and axillary ultrasound, bone scintigraphy, and thoraco-abdominal CT [7]. If clinical examination and/or imaging does not indicate nodal involvement, sentinel lymph node biopsy is the gold standard for axillary surgical approach in women with operable breast cancer. Axillary dissection has been the standard surgical treatment in breast cancer patients for several years, but axillary management has become less invasive, since studies revealed that sentinel lymph node biopsy and targeted axillary dissection have become equal to axillary dissection in clinically node negative and also in certain cases of nodal positive patients regarding local control and prognosis and come with a significantly reduced morbidity [8, 9]. Nevertheless, some studies showed that even in the case of a negative sentinel lymph node biopsy locoregional lymph node metastases are possible [10, 11]. Therefore, highly accurate imaging especially of locoregional lymph nodes becomes even more important in terms of treatment planning.

Involvement of movable axillary lymph nodes is classified as cN1, but it proceeds with involvement of fixed axillary lymph nodes to cN2a, with internal mammary lymph node involvement to cN2b, with infraclavicular involvement to cN3a, with simultaneous axillary and internal mammary lymph nodes to cN3b, and with supraclavicular lymph node involvement to cN3c [12]. This reflects the worsening of prognosing with progression of lymphatic metastasis. Several studies have shown patients with supraclavicular lymph node metastases to have a worse prognosis than patients with nodal involvement limited to axillary levels; nevertheless, TNM classification indicates that patients with supraclavicular lymph node metastases still have a better prognosis than patients with distant metastases [13], other than presumed first.

Although axillary ultrasound is of high value for the detection of axillary lymph node metastases, its value is limited for detection of internal mammary lymph nodes and also for fixed or grouped lymph node metastases. Reliable lymph node assessment especially in these regions is crucial not only for correct N-status and prognosis but also for therapy planning, e.g., in case of internal mammary nodal involvement, enlargement of irradiation field can be considered [14].

So far, studies have shown PET/CT to be superior to conventional imaging methods for nodal staging, but data comparing MRI or PET/MRI with established imaging for nodal staging is still limited. Therefore, the aim of the study was to compare the diagnostic performance of [^18^F]-FDG PET/MRI, MRI, and CT in exact nodal staging in patients with newly diagnosed breast cancer.

## Material and methods

### Patients and inclusion criteria

Patients with therapy-naive invasive breast cancer were included in the study if at least one of the following criteria for elevated risk for distant metastasis was present: (1) newly diagnosed, treatment-naive T2 tumor or higher T stage or (2) newly diagnosed, treatment-naive triple-negative tumor of every size or (3) newly diagnosed, treatment-naive tumor with molecular high risk to set elevated risk for metastases, according to the ESMO guidelines [15]. Patients were included in the study between March 2018 and September 2020. This prospective, double-center study was approved by the local ethics committees (study number 6040R and study number 17–7396-BO). Prior to enrolment, a written informed consent form was signed by all patients. Exclusion criteria were former malignancies in the past 5 years, contraindications for MRI or contrast agents, and pregnancy or breast feeding. Some of the patients were reported before but with different objectives [16, 17].

### (PET/)MRI protocol

All patients underwent (PET/)MR imaging on an integrated 3-T hybrid PET/MRI system (Biograph mMR, Siemens Healthcare, Erlangen, Germany). Patients fasted for 6 h prior to the PET/MRI examination to ensure blood glucose levels were < 150 mg/dl. A weight-adapted dose of [^18^F]-FDG (4 MBq/kg body weight) was intravenously injected 1 h prior to examination. (PET/)MRI was conducted from head to mid-thigh in supine body position for staging purpose. Thoracal sections from whole-body imaging were analyzed for nodal staging.

PET images were reconstructed using the iterative ordered-subset expectation maximization (OSEM) algorithm, 3 iterations and 21 subsets, a Gaussian filter with 4-mm full width at half maximum (FWHM), and a 344 × 344 image matrix. For MR-based attenuation correction of the patient tissues, a two-point (fat, water) coronal 3D-Dixon-VIBE sequence was acquired to generate a four-compartment model (background air, lungs, fat, muscle).

The whole-body MRI protocol comprised the following sequences:

1. A transverse T2-w half Fourier acquisition single shot turbo spin echo (HASTE) sequence in breath-hold technique with a slice thickness of 7 mm (TE 97 ms; TR 1500 ms; turbo factor (TF) 194; FOV 400 mm; phase FOV 75%; acquisition matrix 320 × 240 mm; in plane resolution 1.3 × 1.3 mm; TA 0:47 min / bed position)
2. A transversal diffusion-weighted (DW) echo-planar imaging (EPI) sequence in free breathing with a slice thickness of 5.0 mm (TR 7400 ms; TE 72 ms; *b*-values: 0, 500, and 1000 s/mm^2^, matrix size 160 × 90; FOV 400 mm × 315 mm, phase FOV, 75%; GRAPPA, acceleration factor 2; in-plane resolution 2.6 × 2.6 mm; TA 2:06 min / bed position)
3. A fat-saturated post-contrast transverse 3-dimensional volumetric interpolated breath-hold examination (VIBE) sequence with a slice thickness of 3 mm (TE, 1.53 ms; TR, 3.64 ms; flip angle 9^°^; FOV 400 × 280 mm; phase FOV 75%; acquisition matrix 512 × 384, in-plane resolution 0.7 × 0.7 mm; TA 0:19 min / bed position)

### CT

CT examinations were performed on dedicated CT scanner (Siemens Flash, Siemens Somatom AS, Siemens Healthineers, Erlangen, Germany). Iodinated contrast medium was administered intravenously 70 s before the scan. CT was acquired using the manufacturer-supplied dose reduction CareKV and CareDose 4D.

### Image analysis

Two experienced radiologists and a nuclear medicine specialist with large experience in hybrid imaging and especially PET/MR reading independently analyzed all (PET/)MRI datasets in random order utilizing a Osirix Workstation (Pixmeo SARL, Bernex, Switzerland). Readers were aware of the breast cancer diagnosis but were blinded to patients’ history and identity and to results of distant metastasis. To avoid recognition bias, datasets were evaluated separately with a reading intermission of 4 weeks. Discordant readings were resolved in consensus reading. For every patient, each lymph node station (axillary I–III, supraclavicular, internal mammary chain) was evaluated separately for presence or absence and quantity of lymph node metastasis. Lymph node stations are defined according to the 8th edition of TNM classification as follows: Level I is found lateral from the lateral border of the M. pectoralis minor, level II extends from the lateral to the medial border and posterior to the muscle, while level III extends medial the medial border of the M. pectoralis minor and under the clavicle. Supraclavicular lymph nodes are found above the clavicle and medial from the M. sternocleidomastoideus [18], while internal mammary lymph nodes are located near the internal mammary vessels next to the sternum, mostly in the 1st–6th intercostal space [19, 20].

If applicable, morphologic features for the diagnosis of axillary and supraclavicular lymph node metastases were as follows: (a) short-axis diameter > 10 mm, (b) irregular margin, (c) inhomogeneous cortex, (d) perifocal edema, (e) absent fatty hilum, (f) asymmetry in comparison to contralateral site, (g) contrast media enhancement in comparison to surrounding and to contralateral lymph nodes, and (h) blurred nodal border. Lymph nodes in internal mammary chain were rated suspicious, when 2 or more lymph nodes were > 6 mm [21] or when increased tracer uptake was observed. In PET/MRI, in addition to the previously mentioned morphologic criteria, a tracer uptake above the direct background and the surrounding lymph nodes was considered a sign of malignancy. To measure SUVmax and SUVmean, a manually drawn region of interest was placed around the respective lymph node [22–24]. In accordance with previous publications reporting the superiority of PET uptake over morphology, also small and/or morphologic unsuspicious nodes were rated as malignant when showing elevated FDG uptake [25].

### Reference standard

Histopathology of axillary lymph nodes served as reference standard for patient-based analysis in all patients. Sentinel lymph node biopsy or axilla dissection was used as reference standard, if available. Otherwise, pretherapeutic ultrasound-guided core needle biopsy was used as reference standard. If no sufficient pretherapeutic sampling of lymph nodes was available, sentinel lymph node excision or axilla dissection after neoadjuvant systemic therapy was used as reference standard. For this, additional histopathological preparations were evaluated, using focal fibrosis or focal necrosis as a retrospective indicator for previously vital lymph node metastasis [26, 27]. For lesion-based analysis, all histopathological samples as well as results of follow-up imaging (if available) and clinical follow-up were taken into account to determine the reference standard. Reduction in diameter following treatment was regarded as a sign of malignancy. Also, lesions with increasing size and those with enduring and increasing changes listed above (irregular margin, inhomogeneous cortex, perifocal edema, absent fatty hilum, asymmetry in comparison to contralateral site, contrast media enhancement, and blurred nodal border) were considered malignant.

### Statistics

SPSS Statistics 26 (IBM Corp., Chicago, IL, USA) was used for statistical analysis. A *p*-value < 0.05 was considered statistically significant. Data are presented as mean ± standard deviation. A McNemar test was used to assess differences in lesion detection on a patient-based analysis and on a lesion-based analysis. Based on the reference standard for patient-based analysis, sensitivity, specificity, positive predictive value, negative predictive value, and accuracy were calculated. Because of their location in internal mammary chain, four PET positive, but morphologically not detectable, lymph nodes could not be saved histopathologically. In morphological follow-up imaging, these nodes were still not detectable; therefore, no reliable reference standard was available. To address this insoluble problem, the diagnostic performance was calculated for two different scenarios, on the one hand that PET/MRI rated these lymph nodes falsely positive and MRI as well as CT rated these lymph nodes correctly negative and vice versa.

## Results

### Patient population and reference standard

**Fig. 1:**
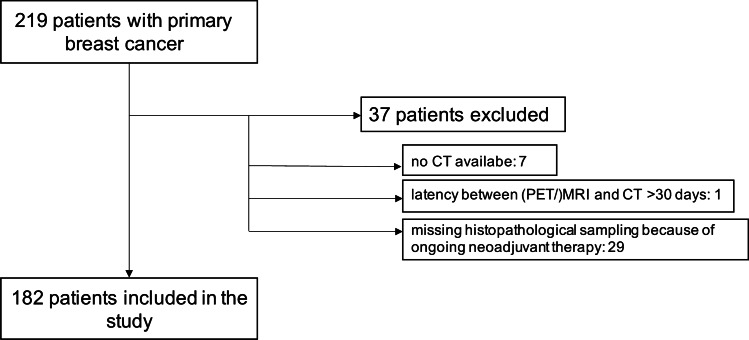
Patient flow diagram

In this study, a total of 182 patients (mean age 52.7 ± 11.9 years) were prospectively included (Fig. 1). For patient demographics and primary tumor characteristics, see Table 1. For all patients, dedicated chest/thorax/pelvis CT, whole-body MRI, and whole-body [^18^F]-FDG PET/MRI (mean activity 278.53 ± 63.95 MBq) were available.

**Table 1:** Patient demographics and primary tumor characteristics for the 182 included patients (three with bilateral tumors)

Total patients		182
Sex		182 female
Mean age (± standard deviation)		52.7 ± 11.9 years
Menopause status		
Pre		84
Peri		13
Post		85
Ki67		
Positive >14%		169
Negative <14%		16
Progesterone status		
Positive		118
Negative		67
Estrogen status		
Positive		134
Negative		51
HER2neu expression		
0		66
1+		58
2+		30
3+		31
Tumor grade		
G1		7
G2		99
G3		79
Histology		
NST		156
Lobular invasive		19
Other		10
TNM staging		
Tumor	T1	65
	T2	106
	T3	9
	T4	5
Nodus	N0	109
	N1	44
	N2	12
	N3	17
Metastases	M0	171
	M1	11

According to the reference standard, 109/182 patients (59.9%) were nodal negative and 73/182 patients (40.1%) were nodal positive. In 93/182 patients (51.1%), histological sampling of lymph nodes was conducted before systemic therapy (52 axillary core needle biopsies, 32 sentinel lymph node excisions, 9 axilla dissections), and in 89/182 patients (48.9%), sampling was conducted after systemic therapy (86 sentinel lymph node excision, 3 axillary dissections). A total of 194 lymph node metastases were detected in all three imaging modalities. In 104 patients, additional MRI follow-up imaging was used to confirm the reference standard.

### Patient-based analysis and diagnostic performance

On a patient-based analysis, PET/MRI detected 60/73 (82.2%) nodal positive (N^+^) patients while classifying 13 patients as false negative (missing 17.8% of N^+^ patients). MRI correctly detected 51/73 (69.9%) N^+^ patients while classifying 22 patients as false negative (missing 30.1% of N^+^ patients) and CT detected 46/73 (63.0%) N^+^ patients while classifying 27 patients as false negative (missing 37.0% of N^+^ patients).

No additional patient was classified as N^+^ in CT or MRI compared to PET/MRI. Seven additional patients were correctly staged N^+^ in MRI but were missed in CT, while 3 other patients were correctly staged N^+^ in CT but were missed in MRI. In four patients, one single lymph node was suspicious for metastasis in PET/MRI due to elevated tracer uptake, but not in MRI or CT. Because of their location (a single internal mammary lymph node in three patients and a interpectoral lymph node in one patient), these lymph nodes could not be saved histopathologically, but reference standard (sentinel lymph node biopsy) was negative for all four patients. As described before, two different scenarios were calculated for sensitivity, specificity, positive predictive value (PPV), negative predictive value (NPV), and accuracy for each modality to solve this problem (see Table 2). On a patient-based analysis, differences between PET/MRI and MRI and between PET/MRI and CT were statistically significant (each *p* < 0.0001). Differences between MRI and CT were statistically non-significant (*p* = 0.7893).

**Table 2:** Diagnostic performance of PET/MRI, MRI, and CT on a patient-based analysis in differentiating N^+^ and N^−^ status, when all 4 lymph nodes, that could not be saved histopathologically are (A) rated false positive in PET/MRI and true negative in MRI and CT or (B) rated true positive in PET/MRI and false negative in MRI and CT

	Sensitivity	Specificity	PPV	NPV	Accuracy
**A)**					
PET/MRI (95% CI)	82.43% (71.83 to 90.30%)	86.36% (78.51 to 92.16%)	80.26% (71.52 to 86.82%)	87.96% (81.61 to 92.33%)	84.78% (78.76 to 89.64%)
MRI (95% CI)	69.86% (58.00 to 80.06%)	95.45% (89.71 to 98.51%)	91.07% (81.04 to 96.05%)	82.68% (77.05 to 87.15%)	85.25% (79.26 to 90.05%)
CT (95% CI)	63.01% (50.91 to 74.03%)	96.36% (90.95 to 99.00%)	92.00% (81.22 to 96.83%)	79.70% (74.38 to 84.15%)	83.06% (76.83 to 88.19%)
**B)**					
PET/MRI (95% CI)	83.12% (72.86 to 90.69%)	89.52% (82.03 to 94.65%)	85.33% (76.72 to 91.13%)	87.85% (81.43 to 92.26%)	86.81% (81.02 to 91.36%)
MRI (95% CI)	66.23% (54.55 to 76.62%)	95.24% (89.24 to 98.44%)	91.07% (81.03 to 96.06%)	79.36% (73.72 to 84.06%	82.97% (76.70 to 88.12%)
CT (95% CI)	59.74% (47.94 to 70.77%)	96.19% (90.53 to 98.95%)	92.00% (81.21 to 96.83%)	76.52% (71.23 to 81.09%)	80.77% (74.28 to 86.22)

*PPV* positive predictive value, *NPV* negative predictive value

### Lesion-based analysis

**Fig. 2:**
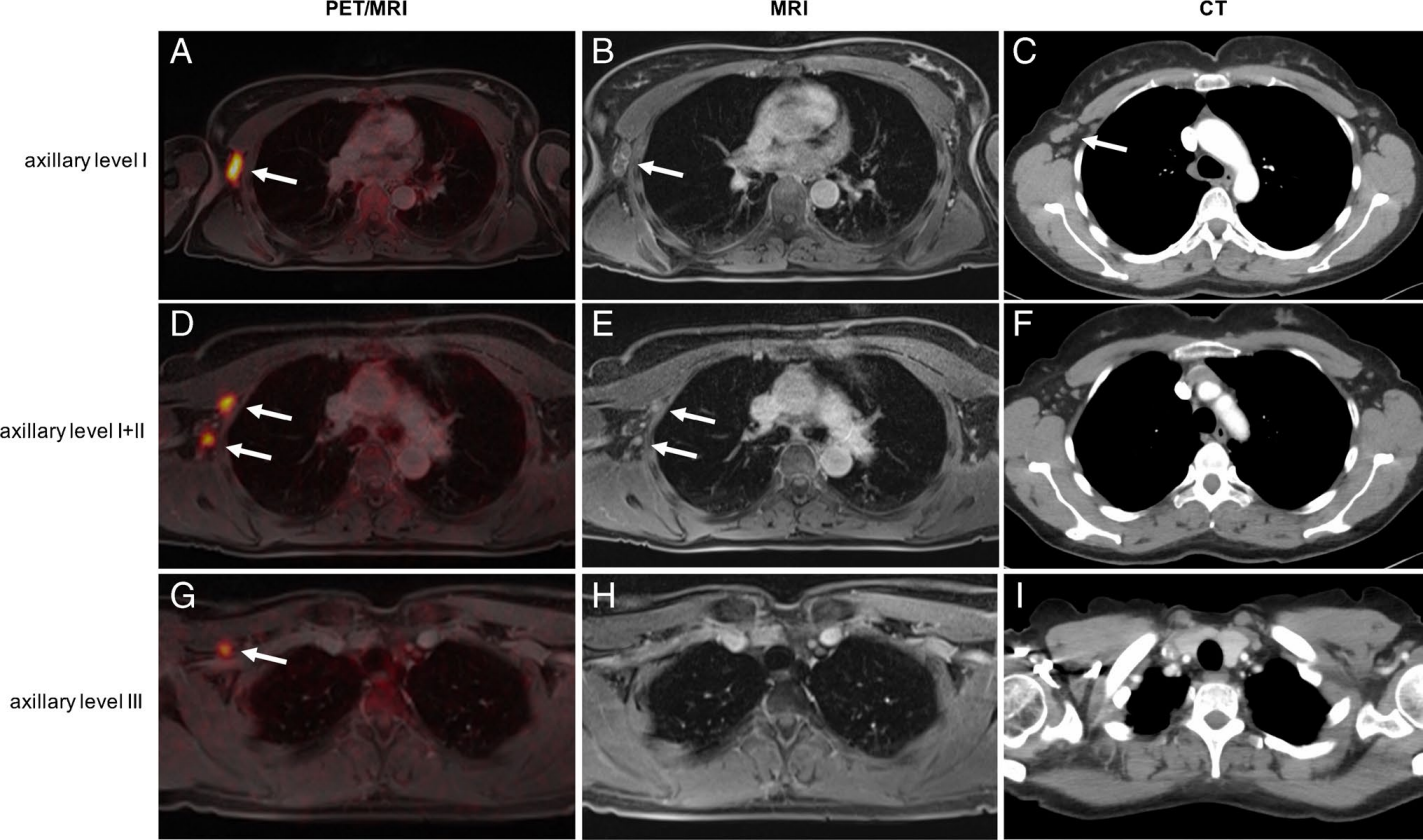
Lymph node metastases (white arrows) detected in different imaging modalities. **A**–**C** Lymph node metastasis in axillary level I detected in PET/MRI (**A**), MRI (**B**), and CT (**C**). **D**–**F** Lymph node metastases in axillary levels I and II detected in PET/MRI (**D**) and MRI (**E**), but not in CT (**F**). **G**–**I** Lymph node metastasis in axillary level III detected in PET/MRI (**G**), but not in MRI (**H**) and CT (**I**)

CT detected 104 lymph node metastases, 69 of them in axillary level I, 34 in axillary level II, and one in internal mammary chain. MRI detected 123 lymph node metastases, 80 of them in axillary level I, 38 in axillary level II, two in axillary level III, and three in internal mammary chain. PET/MRI detected 193 lymph node metastases, 102 of them in axillary level I, 61 in axillary level II, 13 in axillary level III, one supraclavicular lymph node, and 16 internal mammary chain lymph nodes (Table 3). Overall differences in lesion-based detection rates were statistically significant (PET/MRI vs. MRI, *p* < 0.0001; PET/MRI vs. CT, *p* < 0.0001; MRI vs. CT, *p* = 0.015) (for an example, see Fig. 2). Subgroup analysis showed that differences between PET/MRI vs. MRI and PET/MRT vs. CT were statistically significant for each lymph node station. Differences between MRI and CT were statistically significant only for axillary level I, while differences for axillary level II + III, supraclavicular, and internal mammary stream were statistically non-significant (Table 4).

**Table 3:** Number of suspicious lymph nodes in the different locations

	**CT**	**MRI**	**PET/MRI**
Axillary level I	69	80	102
Axillary level II	34	38	61
Axillary level III	0	2	13
Supraclavicular	0	0	1
Internal mammary stream	1	3	16
Total	104	123	193

**Table 4:** Comparison of numbers of detected lymph node metastases per location with respective *p*-values

	**MRT vs. PET/MRT**	**CT vs. PET/MRT**	**MRT vs. CT**
Axillary I	< 0.0001	< 0.0001	0.0291
Axillary II	< 0.0001	< 0.0001	0.3865
Axillary III	0.0026	0.0015	0.4795
Supraclavicular*	MRT: no; PET/MRT: yes	CT: no; PET/MRT: yes	MRT: no; CT: no
Internal mammary stream	0.0009	0.0003	0.4795

^*^Only PET/MRI detected one supraclavicular lymph node metastasis, which was not detected by MRI and CT

Compared to MRI, PET/MRI leads to an upstaging of nodal status in 30 patients, and compared to CT, PET/MRI led to an upstaging in 41 patients. No downstaging was observed in PET/MRI compared to that in MRI or CT. Compared to CT, MRI led to an upstaging of nodal status in 15 patients and to a downstaging in 6 patients.

## Discussion

This study demonstrates the diagnostic superiority of [^18^F]-FDG PET/MRI over MRI and CT in determining the correct nodal status in axillary (levels I–III), supraclavicular, and internal mammary lymph nodes in patients with newly diagnosed breast cancer. On a patient-based analysis, PET/MRI correctly detected nodal involvement in significantly more patients and on a lesion-based analysis, PET/MRI detected significantly more lymph node metastases per lymph node station. As no adequate reference standard was available for a total of 4 lymph nodes along the internal mammary chain, two different scenarios were calculated: However, if the 95% CI intervals of both calculation bases are taken as a basis, it can be assumed that PET/MRI is superior to MRI with regard to overall accuracy. This is pooled for PET/MRI at 78.76 to 91.36% and for MRI at 76.6 to 85.25%.

Reliable and exact detection of nodal positive disease is crucial in breast cancer, not only for therapy planning, but also in terms of prognosis.

As several guidelines for breast cancer recommend sonography and CT for nodal staging of primary breast cancer patients [7], this is the imaging gold standard for assessment of nodal involvement so far. But studies have shown limited sensitivity of both imaging modalities: Alvarez et al. have postulated that sonography is of limited use to exclude nodal involvement due to its only moderate sensitivity [28]. Studies on the detection accuracy of lymph node metastases in CT are limited. Individual studies have shown that sensitivity of CT for the detection of axillary nodal involvement in breast cancer patients is 76.9%, although a short-axis diameter of 5 mm was set as the cutoff value for suspicious lymph nodes [29]. In our study, CT showed even lower sensitivity with only 63%. Due to its high sensitivity and high negative predictive value, some studies even suggest replacing sentinel lymph node biopsy by MRI [30, 31]. But compared to our study, dedicated axillary protocols were applied and/or lymph node-specific contrast agents were used. Contrary to these protocols, we examined the value of MRI in an everyday clinical setting and nearly similar to CT, MRI revealed a limited sensitivity with about 70%.

Studies with small patient cohorts even show the added value of PET/MRI in nodal staging of primary breast cancer patients, compared to conventional imaging methods as ultrasound and MRI [32, 33].

As PET/CT is an extensively studied imaging modality, various studies exist on the diagnostic performance of the detection of nodal involvement in primary breast cancer patients. In different prospective studies, a sensitivity from 56 to 77% is described [34–36]. For PET/CT, it is reported that false negative axillae had significantly smaller and fewer tumor positive lymph nodes than true positive axillae [37]. These findings go hand in hand with our study, demonstrating a sensitivity of PET/MRI in detecting nodal positive patients of over 80%.

Quantity of affected lymph nodes is of importance especially for planned radiotherapy. If > 3 axillary lymph nodes are affected, the radiation field would include the infraclavicular and supraclavicular lymph nodes [38, 39]. Imaging is particularly important if lymph nodes other than the later removed sentinel lymph node are conspicuous in imaging. In addition, studies have shown that the sentinel lymph node can also be found outside the axilla, which entails the risk of underclassifying patients in whom a sentinel lymph node biopsy only includes axillary lymph nodes [40]. Other studies have shown that sentinel lymph nodes can also be located outside of predefined lymph node stations, the so-called interval nodes [5]. In this case, too, the removal of only axillary lymph nodes as sentinel lymph node biopsy carries the potential risk of false negativity. Therefore, in sentinel lymph node biopsy, preoperative imaging should always be considered with regard to conspicuous lymph nodes outside the axilla. Literature indicates that PET/CT can provide information about extra-axillary nodal involvement better than conventional imaging methods [41] and that the diagnosis of extra-axillary lymph node metastases with clinically well-established methods shows a lack of sensitivity. This is in line with our study, demonstrating the superiority of PET/MRI over MRI and CT in detecting lymph node metastases not only for axillary levels I–III, but also for supraclavicular and internal mammary nodes. For example, in our study, PET/MRI was the only imaging modality, which was able to detect supraclavicular nodal metastatic disease. Staging of internal mammary lymph nodes is controversial because there is no existing cutoff size for pathological enlargement and presence of internal mammary lymph nodes can also be observed in healthy individuals [42]. Therefore, especially increased metabolic activity in hybrid imaging is indicative for nodal metastasis [43]. This is underlined by the results of this study, as in some cases tracer uptake was seen in internal mammary chain without a certain morphological correlate. The therapeutic consequence of the additional internal mammary lymph node metastases detected in PET/MRI lies primarily in the extension of the radiation field with inclusion of the internal mammary chain [44], since most patients are treated neoadjuvantly anyway and resection of internal mammary lymph nodes is very invasive and bears a high risk of complications.

Our study has some limitations. First of all, not all suspect lymph nodes are proven histopathologically. Mostly, sentinel lymph node or ultrasound-guided biopsies were taken from axillary level I or II. Also in the case of axillary dissection, sampling is limited to axillary levels I and II. In our study, supraclavicular lymph nodes and lymph nodes of the internal mammary chain were not proven histopathologically. Consequently, diagnostic performance could not be calculated for lesion-based analysis, but for patient-based analysis only. Furthermore, only one patient had a suspicious supraclavicular lymph node, but this is in line with literature reporting only 1–4% of patients without distant metastasis having supraclavicular lymph node metastasis [45]. Hence, statistical analysis for supraclavicular lymph nodes could not be made.

In conclusion, our prospective study demonstrates that PET/MRI localizes lymph node metastases with higher detection rate and accuracy than MRI and CT and that it can reliably be used for nodal staging in primary breast cancer patients. PET/MRI is superior not only in axillary levels I and II, which are commonly covered by sentinel lymph node biopsy and axillary dissection, but also in axillary level III, supraclavicular, and internal mammary lymph nodes, which may have impact on modified axillary dissection or enlargement of irradiation fields. Although diagnostic superiority could be shown, effects on patient outcome have to be evaluated in further prospective studies.

## Data Availability

The authors ensure that all data and materials as well as software application support their published claims and comply with field standards. Code availability Not applicable.
